# OSCA: a comprehensive open-access system of analysis of posterior capsular opacification

**DOI:** 10.1186/1471-2415-6-30

**Published:** 2006-08-23

**Authors:** Tariq M Aslam, Niall Patton, Christopher J Rose

**Affiliations:** 1 Princess Alexandra Eye Pavilion, Chalmers St, Edinburgh, UK; 2 Division of Image Science and Biomedical Engineering, Manchester University, Oxford Road, UK

## Abstract

**Background:**

This paper presents and tests a comprehensive computerised system of analysis of digital images of posterior capsule opacification (PCO). It updates and expands significantly on a previous presentation to include facilities for selecting user defined central areas and for registering and subsequent merging of images for artefact removal. Also, the program is compiled and thus eliminates the need for specialised additional software. The system is referred to in this paper as the open-access systematic capsule assessment (OSCA). The system is designed to be evidence based, objective and openly available, improving on current systems of analysis.

**Methods:**

Principal features of the OSCA system of analysis are discussed. Flash artefacts are automatically located in two PCO images and the images merged to produce a composite free from these artefacts. For this to be possible the second image has to be manipulated with a registration technique to bring it into alignment with the first. Further image processing and analysis steps use a location-sensitive entropy based texture analysis of PCO. Validity of measuring PCO progression of the whole new system is assessed along with visual significance of scores. Reliability of the system is assessed.

**Results:**

Analysis of PCO by the system shows ability to detect early progression of PCO, as well as detection of more visually significant PCO. Images with no clinical PCO produce very low scores in the analysis. Reliability of the system of analysis is demonstrated.

**Conclusion:**

This system of PCO analysis is evidence-based, objective and clinically useful. It incorporates flash detection and removal as well as location sensitive texture analysis. It provides features and benefits not previously available to most researchers or clinicians. Substantial evidence is provided for this system's validity and reliability.

## Background

Cataract extraction is the most common ophthalmic surgical procedure and posterior capsule opacification (PCO) remains the most common post-operative cause of morbidity[1]. There are many factors known to influence PCO[2] and there is abundant research into prevention and treatment of this condition. Objective research requires a reliable and valid outcome measure[3]. It is essential for unbiased and incontrovertible scientific progress to have an open, accessible system that can freely be used in the scientific community. However, there is currently no consensus on an optimal quantification method for PCO analysis. There are many competing systems with varying degrees of validity and objectivity. In particular, the POCO[4] and AQUA[5] systems demonstrate elaborate and appropriate algorithms for analysis but are not openly available. They do not incorporate eccentricity of PCO into calculations, which we believe improves clinical validity in terms of correlation of scores with visual deficit. The POCO system does not evaluate PCO severity. Another system, EPCO[6], has been assessed for evidence of construct validity but is subjective. The POCOman system[7] is also subjective and is not convincing for analysis of PCO in terms of measuring progression or visual significance[8].

In a previous paper we presented our own system of PCO analysis[9]. However it was limited in that it did not incorporate any objective mechanism for complete removal of flash photography. It required prior processing to segment out specific sized capsular areas of interest and also required the images to have prior subjective removal of flash artefact using separate software. These characteristics limited the objectivity of analysis. The previous system also required the user to have the Matlab^® ^(Natwick, Mass, USA) numerical programming environment already installed, limiting its availability to the research population. The system presented today (OSCA) corrects these weaknesses and is complete, automated and easily usable.

It is based upon current evidence on the visual significance of PCO and contains the best features of modern systems that have already made advances in PCO analysis. We developed our program independently, and created new and novel algorithms to optimise validity and objectivity. We designed it to be the optimum program for analysis. Having the advantage of being open access will also benefit future PCO research.

## Methods

### Development of software/Programming

Software design and programming was performed by one author (TA) and the system is referred to as the Open-access Systematic Capsule Assessment (OSCA) in this paper. Informed consent was obtained from all patients involved and the study was approved by local ethics committee, in accordance with the declaration of Helsinki.

The main challenge involved in the development of the new system of analysis from previous formats was in the incorporation of a method of removing flash artefact from images. Some systems of PCO analysis merely exclude areas of flash from subsequent analysis[4]. However this alone is not ideal as the potential area of PCO under the flash remains unaccounted for. Findl et al. [10] however, have published a mechanism by which two images containing spoilt flash areas in different regions are overlapped to form a composite in which all of the image represents unspoiled PCO. Thus areas of flash are replaced by corresponding areas of PCO from another image of the patient's capsule. Other authors have used similar techniques of flash removal in a non-automated manner[6,11]. An automated model of flash removal was incorporated into the OSCA system, detailed below. We created our own algorithms to optimise sensitivity and specificity of flash detection.

Each image is photographed twice, from different angles, with the light reflection in a different area of the image. The two images are then aligned using image registration techniques. The aim of image registration is to apply spatial transformations to the input images to bring them into alignment with the base image. The input images may be somewhat misaligned due to the different camera angles required to induce flash in different areas. The details of the exact alignment algorithm are calculated after the user identifies four pairs of points that should exactly correspond in the input and base images. A spatial mapping is inferred from the positions of these control points [12]. The input image is then transformed into alignment with the base image so that direct comparisons can be made. The OSCA system then identifies areas regarded as bright flash. These areas are segmented using a combination of threshold techniques that were found in iterative testing to produce the most valid results. The intensities of the final greyscale images were not solely used to identify flash, but also individual values and relationships that were found between the red, green and blue channels of the RGB image in areas of flash. These initial core areas are further processed by a combination of erosion and dilation morphological operators to expand the effective area coded as artefact flash. Such mathematical morphological algorithms are able to process images based upon the 'shape' of features in the image[13]. The end result is that the system is able to segment areas of flash along with the immediate surrounding areas of pixels that may also have been affected by the core artefactual light.

In the final stage of merging, portions of flash-spoiled image from the main photo are replaced by the exact corresponding but unspoiled regions from the second, registered image, creating a final composite free of unwanted reflections. AnotAAAher new feature is that the user is able to specify the size of capsular area to be analysed. The size in pixels for a feature of known true size, such as a 5 millimetre IOL optic diameter, is first calculated on an image where it is visible. Number of pixels that correspond to any set number of central millimetres can thus be deduced. Of course this may be different from one photographic/computer setup to another. At the end of the processing tree the user has the option of segmenting out only this pixel diameter for analysis. This final, composite, resized image is then analysed by the previously published texture analysis algorithm to calculate overall PCO score[9].

A major benefit of the new PCO analysis system is that it has been compiled for use onpersonal computers running the Microsoft Windows XP^® ^operating system. This means that the PCO analysis system can be distributed and easily installed on end-users' computers. End-users no longer require a Matlab^® ^license or installation, removing both technical and financial impediments to the adoption of the system. (Note however that researchers wanting to alter the software will still need a Matlab^® ^license and installation.) All of the program's source code is available from TA on request.

### Testing for validity

#### Visual relevance of OSCA (convergent validity)

Evidence for convergent validity involved study of thirty-five patients that were recruited after having been referred for potential Nd: YAG capsulotomy. One eye of each patient was studied. Full details of the experiment and image acquisition methods are described previously[14].

On attendance, patients had contrast sensitivity tested with Pelli-Robson charts. Patients were dilated and the posterior capsules photographed with Imagenet^® ^digital photography system and Topcon^® ^camera system at standardised settings, with eyes positioned so that flash appeared in different regions in two separate images. Images were subsequently stored onto disc. Each patient underwent Nd: YAG laser capsulotomy via a set protocol by one surgeon. Patients returned one week later and had further vision testing. They were again dilated and posterior capsules photographed at standardised settings. For this study, all images were analysed by OSCA only, by one examiner, with no other pre-processing and PCO score recorded.

For evidence of convergent validity, results of PCO analysis were correlated with vision. Specifically, the difference in PCO quantification by the OSCA system before and after Nd: YAG capsulotomy was compared to improvement in both LogMAR distance visual acuity and contrast sensitivity for each patient using regression analysis. A graphical representation of results was analysed as well as the indices of regression analysis.

For the purposes of validity and reliability studies and to maintain standardisation, the area taken for all our analyses comprised of the area within the dilated pupil border. The system however does alternatively allow for a user defined central area to be used.

#### Measurement of progression by OSCA system

In order to test the system's ability to measure progression, digital images of 21 patients a month after cataract extraction, who were deemed to have little significant PCO were analysed. Also analysed were images of 21 patients 12–18 months after cataract surgery who were deemed on inspection to have shown a significant degree of PCO development since their cataract extraction. In these patients PCO was visible but mainly peripheral PCO and mild. One would expect a model system to produce values reflecting very low levels of PCO in patients within a month of surgery, and for those values to be significantly less than those in patients 12–18 months after cataract extraction, in whom progression is visible. The results for the OSCA system were plotted and analysed to assess whether findings were significantly different in the two groups.

### Testing for reliability

To assess interobserver reliability of the OSCA system, two trained observers performed analysis on a sample of 12 images. These images included both pre- and post- Nd: YAG capsulotomy, as well as a selection of images with no PCO. The ICC (intraclass correlation coefficient) was calculated, as well as the coefficient of repeatability. In addition, a Bland-Altman plot to graphically examine the repeatability was plotted.

## Results

### Use of final system

The final system operates through a user-friendly graphical user interface (GUI) after installing basic program files [see Additional files 1–8]. The user works his way down a sequence of buttons to initiate each step of the processing and analysis. The system requires the user to input an image, and loads that image for analysis. The second image is also loaded and both images appear on screen. The second image is then registered in relation to the first – the user is required to click on a key point in the first image and its exact corresponding point in the second. This is repeated four times to create reference values for a subsequently calculated transformation. The transformed image is also displayed on screen. The user then delineates precisely the region of capsule that is to be measured. In the validity experiments presented here, the area within a pupil's borders were used for analysis. Alternately the system allows for specified sizes of central areas to be measured, with the centre of the image defined by the geometric centre of the pupil borders. After this stage the user proceeds to the 'Remove Background' and 'Swap Bright Areas' procedures. Finally, clicking on 'Calculate' scores the level of PCO in the final merged, flash corrected, background removed image. The processed images are displayed along with final texture measures.

The range of possible OSCA scores is from 0 (no PCO) to approximately 15 (practical expected maximum). Typical OSCA values for images with very little or no PCO were around 0.5, with noise or incorporation of lens edge and other artefacts artificially raising values slightly. Values for patients that were deemed to warrant laser capsulotomy were typically around 4–5, whilst in our images the maximum recorded amount of PCO measured by OSCA was approximately 12. A typical analysis would take 2–3 minutes to complete.

### Testing for validity

#### Visual relevance of OSCA (convergent validity)

**Figure 1:**
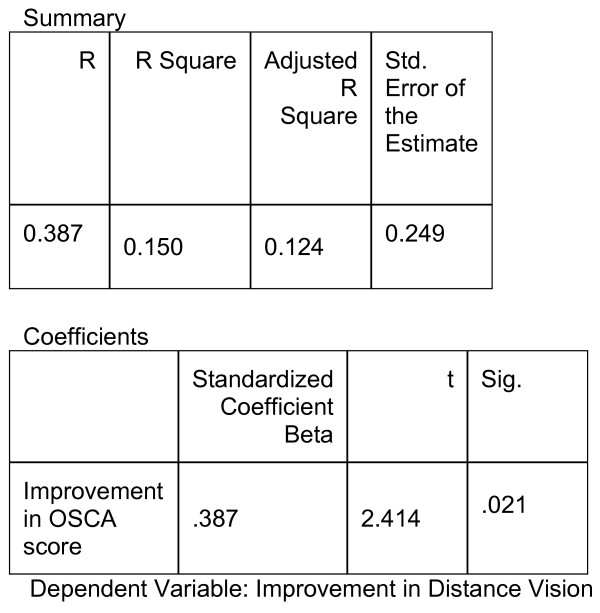
Improvement in distance vision compared to improvements in OSCA score, after Nd:YAG Capsulotomy.

Linear regression was used in assessing the impact of the OSCA score on visual function measured. The first dependent variable to be studied was improvement in best-corrected distance LogMAR visual acuity (DLVA). Linear regression analysis revealed a standardised coefficient of β = 0.387, (p = 0.021, R^2 ^0.150)(Fig. 1)

**Figure 2:**
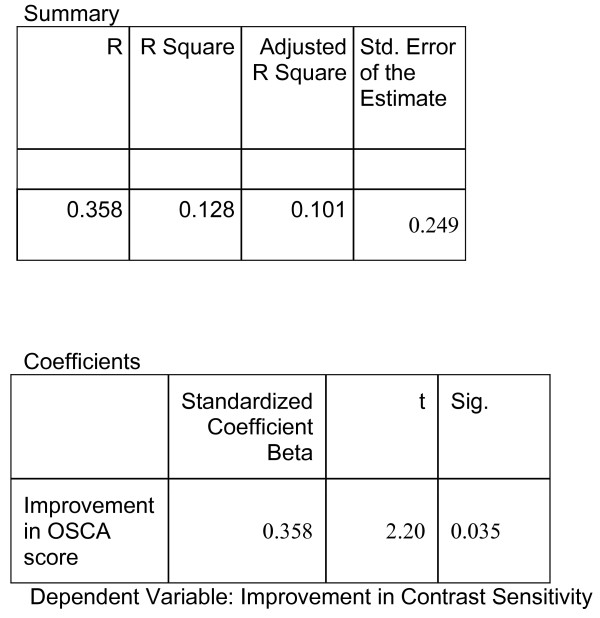
Improvement in contrast sensitivity compared to improvements in OSCA score, after Nd:YAG Capsulotomy.

**Figure 3:**
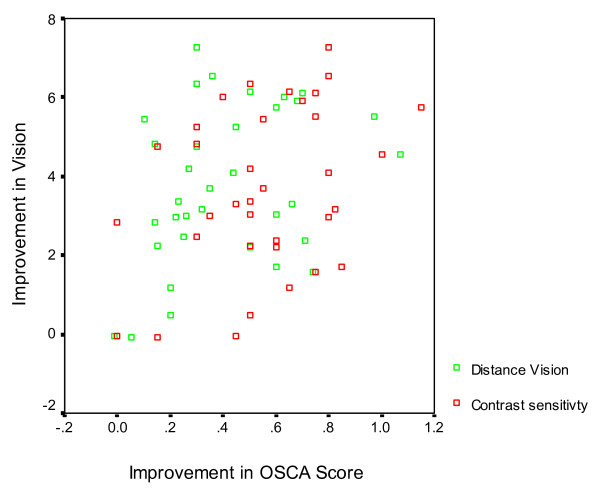
Scatter plot of improvement of OSCA scores versus improvement in contrast sensitivity and distance vision following Nd:YAG capsulotomy.

The second dependent variable to be studied was improvement in contrast sensitivity (CS). Linear regression analysis showed that the standardised coefficient β = 0.358, p = 0.035, R square 0.128 (Fig. 2). A scatter graph demonstrates this correlation of OSCA score with contrast sensitivity and visual acuity (Fig. 3).

#### Progression of PCO over time

**Figure 4:**
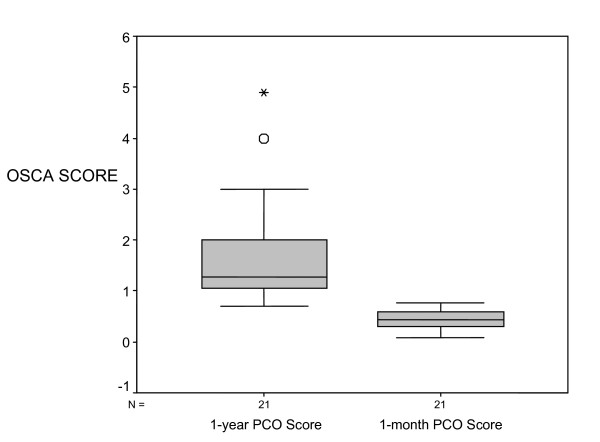
Box plot diagram representing median (mid-line), interquartile range (shaded boxes) and range (whiskers) comparing OSCA scores for PCO one month after PCO compared to 18 months after PCO (p < 0.01).

A boxplot graph of OSCA scores in 21 patients one month after surgery compared to 21 patients 12–18 months post surgery is illustrated in Fig. 4. Analyses of frequency distribution of PCO scores in these two groups reveal normality of data. However variances of the two samples differ. Mann Whitney U test was therefore used to compare scores. The Mann Whitney U test showed significant differences in the two sets of values (p < 0.001).

### Testing for reliability

A total of 12 images were analysed once by two observers. The ICC was 0.997 (95% confidence intervals 0.991 to 0.999). Coefficient of Repeatability was ± 0.38 (i.e. 95% of repeated measures would be expected to be within this margin).

**Figure 5:**
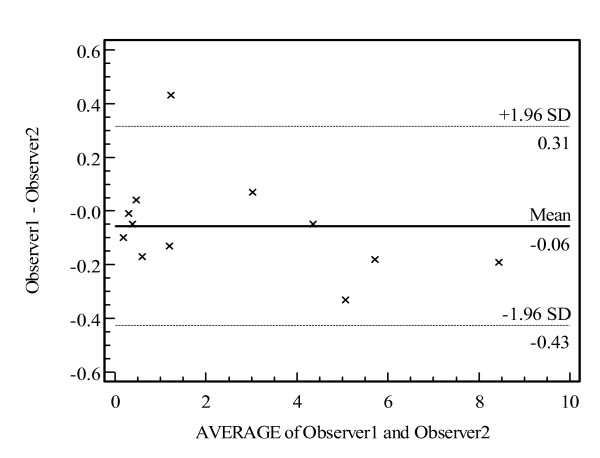
Bland-Altman plot of interobserver reliability for Observer 1 and Observer 2 for the 12 pairs of images. Mean difference was -0.06 and 95% of repeated measurements would be expected to be within the values 0.31 to -0.43.

The "limits-of-agreement" plot (Bland-Altman plot) revealed no systematic bias between observer 1 and observer 2 for the 12 pairs of images (Fig. 5).

## Discussion

Improvements in distance vision and contrast sensitivity after Nd: YAG capsulotomies are shown to be strongly correlated to the entropy score change for PCO in the OSCA system by regression analysis. This represents substantial evidence for convergent validity. We demonstrated this technique for assessing visual relevance of PCO measures in 2003[14]. Such correlation of measures before and after YAG capsulotomy have since become commonly used in assessing visually significant PCO[15,16]. For the purposes of validity and reliability studies and to maintain standardisation, the area taken for all our analyses comprised of the area within the dilated pupil border, though the system does allow for user defined areas to be used instead. In a recent paper, Buehl et al. [17] similarly compared measures of PCO before and after Nd: YAG capsulotomy. They measured only the areas within each patient's pupil size (measured under reading conditions) and found a correlation with visual function. Although not a centrally weighted measure, the AQUA system used in this way shows excellent validity. However, such measurement technique would not be sensitive to small amounts of peripheral PCO.

We have shown that positive weighting towards central visual axis of the OSCA score results similarly in visual significance of scores. However, by using dilated images for all our scores, we are able to use a single centrally weighted PCO score that is not only visually significant, but which also allows the same score and measurement technique to be used to assess smaller amounts of peripheral PCO in early progression as well as larger amounts of advanced PCO. This feature may be important in trials assessing different intraocular lenses and their effect on PCO.

In measurement of progression of PCO over time, PCO scores in 21 patients one month after surgery compared to 21 patients 12–18 months post surgery showed statistically significant differences. It is evident that the OSCA system is able to differentiate between patients with early PCO and no PCO. We chose different patients for both groups. This would challenge the systems ability to provide valid measures against not only random intra-individual variations but also variations such as changes in retinal pigment or IOL borders. Again this evidence is most pertinent for a system that may be used to assess the differences in PCO in two different groups in a randomised trial.

Finally, results from calculation of coefficients of repeatability show that the OSCA system demonstrates excellent reliability. Small variations that do exist occurred when there were slightly different areas chosen for analysis related to poorer images with excessive flash obscured pupil boundaries.

## Conclusion

This paper describes a logical evidence-based system of analysis of PCO which represents an advancement not only on our own previously published system but on other available and exclusive systems of PCO analysis. Furthermore the OSCA system is able to be used by any experimenter with access to a computer running Windows XP. It performs all processing tasks that have been presented in less obtainable systems, including registration-based merging of images. It incorporates novel analytical techniques that are not found in other authors system but which were found to enhance validity. Despite the high level of automation of this new system we are still able to demonstrate evidence for validity as well as reliability. We believe it is the most objective, complete and available system for research into the problem of PCO.

Whilst the presented program in this study advances the currently available systems of PCO analysis, we would expect the system to be updated following future research findings as well as further data from analyses.

## Availability and requirements

The software is available from the author by e mailing him on TAslam@AOL.COM. The reader will be sent a link to a site for downloading all the files, including full instructions on set up and use.

## Competing interests

The author(s) declare that they have no competing interests.

## Authors' contributions

Tariq Aslam devised the system of analysis and did all the software programming. Niall Patton collaborated in testing of the system, in particular the reliability studies and reviewed system assessment. Chris Rose assessed and contributed to the whole paper from an image processing viewpoint. All authors have read and approved the final manuscript.

## Pre-publication history

The pre-publication history for this paper can be accessed here:


